# Thyrotoxic periodic paralysis and chorea: two uncommon neuromuscular complications in an adolescent with newly diagnosed Graves disease

**DOI:** 10.1186/1687-9856-2013-S1-P145

**Published:** 2013-10-03

**Authors:** Pairunyar Nakavachara, Montira Suppakrucha, Surachai Likasitwattanakul

**Affiliations:** 1 Division of Pediatric Endocrinology, Department of Pediatrics, Faculty of Medicine Siriraj Hospital, Mahidol University, Bangkok, Thailand; 2 Division of Pediatric Neurology, Department of Pediatrics, Faculty of Medicine Siriraj Hospital, Mahidol University, Bangkok, Thailand

## 

Both thyrotoxic periodic paralysis (TPP) and choreoathetosis are unusual complications of childhood thyrotoxicosis. We hereby report a boy who presented with both TPP and choreoathetosis at the initial presentation of his Graves disease.

A 14 year-old Thai boy presented with acute generalized proximal muscle weakness and myalgia for 5 hours. Detailed history revealed the increased appetite, heat intolerance and labile mood for 6 months. Physical examination revealed tachycardia, goiter and mild exopthalmos. Generalized proximal muscle weakness and hyporeflexia were noted. Abnormal movement including ballism and mild spooning of hands were also noted. The milk maid sign was positive. The abnormal movement was consistent with choreoathetosis. Labs showed serum T3 > 651 ng/dL, Free T4 > 7.77 ng/dL, TSH < 0.005 uU/mL, K 2.1 mmol/L and CPK 1555 U/L. Autoimmune profile showed high titer of the anti-microsomal antibodies of 1:25000. The final diagnosis of Graves disease with TPP and choreoathetosis were made.

Methimazole and propranolol were started. Intravenous K was administered to correct hypokalemia. Serum K was normalized within 7 hours after K replacement. The patient was able to stand and walk normally on the 2^nd^ day of admission. After 4 weeks of treatment with Methimazole, the thyroid function test was consistent with hypothyroidism, therefore the dose of Methimazole was decreased. The choreoathetosis resolved completely within 4 weeks.

Our case report demonstrates the first pediatric patient with Graves disease who developed combined neuromuscular complications which were TPP and choreoathetosis. There has been a previous case report of a 21-year-old man with Graves disease who had both muscular complication and choreoathetosis. The muscular involvement in that patient was different from our patient. In that particular patient, he developed acute severe thyrotoxic myopathy which involved both bulbar and skeletal muscle.

TPP is an alarming and potentially lethal complication of hyperthyroidism. It usually resolves completely after the normalization of serum K levels. Effective control of hyperthyroidism can prevent the recurrence of TPP. While, choreoathetosis usually resolves after the patient recovers from hyperthyroid state. We should always be aware of Graves disease in any children who present with either muscle weakness or choreoathetosis since the signs and symptoms of Graves disease itself might not be very obvious.

